# Diagnostic efficiency of whole exome sequencing in the search for genetic causes of hereditary diseases in Yugra (West Siberia, Russia)

**DOI:** 10.18699/vjgb-26-52

**Published:** 2026-05

**Authors:** M.Yu. Donnikov, P.A. Suchko, A.V. Morozkina, L.N. Kolbasin, E.A. Popova, S.I. Papanov, Yu.S. Koshevaya, L.G. Danilov, Yu.A. Eismont, O.S. Glotov, L.V. Kovalenko

**Affiliations:** Medical Institute of Surgut State University, KHMAO-Yugra, Surgut, Russia; Research Institute of Obstetrics, Gynecology, and Reproductology named after D.O. Ott, St. Petersburg, Russia Saint Petersburg State University, St. Petersburg, Russia; Medical Institute of Surgut State University, KHMAO-Yugra, Surgut, Russia; Medical Institute of Surgut State University, KHMAO-Yugra, Surgut, Russia KHMAO-Yugra Surgut Regional Clinical Center for Maternity and Childhood Protection, Medical Genetic Counseling Service, Surgut, KHMAO-Yugra, Russia; Medical Institute of Surgut State University, KHMAO-Yugra, Surgut, Russia KHMAO-Yugra Surgut Regional Clinical Center for Maternity and Childhood Protection, Medical Genetic Counseling Service, Surgut, KHMAO-Yugra, Russia; Medical Institute of Surgut State University, KHMAO-Yugra, Surgut, Russia KHMAO-Yugra Surgut Regional Clinical Center for Maternity and Childhood Protection, Medical Genetic Counseling Service, Surgut, KHMAO-Yugra, Russia; Saint-Petersburg State Medical Diagnostic Center (Genetic Medical Center), St. Petersburg, Russia; Saint Petersburg State University, St. Petersburg, Russia; Federal Scientific and Clinical Center of Infectious Diseases of the Federal Medical and Biological Agency, St. Petersburg, Russia; Research Institute of Obstetrics, Gynecology, and Reproductology named after D.O. Ott, St. Petersburg, Russia Federal Scientific and Clinical Center of Infectious Diseases of the Federal Medical and Biological Agency, St. Petersburg, Russia; Medical Institute of Surgut State University, KHMAO-Yugra, Surgut, Russia

**Keywords:** whole exome sequencing, hereditary diseases, new generation sequencing, genetic counseling, diagnostic effectiveness, molecular genetic diagnosis, полноэкзомное секвенирование, наследственные заболевания, секвенирование нового поколения, генетическое консультирование, эффективность диагностики, молекулярная диагностика

## Abstract

Whole-exome sequencing (WES) has revolutionized the diagnostics of hereditary diseases, yet its efficacy varies across populations. Data on the genetic architecture of rare hereditary disorders in many Russian regions, including the ethnically diverse Khanty-Mansi Autonomous Okrug (Yugra) are scarce. The aim of this study was to evaluate the diagnostic yield of WES for identifying genetic variants associated with hereditary disorders in this ethnically heterogeneous population. The study involved 286 probands with suspected hereditary disorders observed by regional geneticists in the years 2021–2024. WES was performed on the DNBSEQ-G50 platform (MGI, China). Bioinformatic analysis included variant calling and annotation using population databases and pathogenicity prediction tools. Identified variants were classified according to ACMG/Russian Medical Genetics Society guidelines and correlated with clinical phenotypes. Molecular genetic diagnoses were categorized as definitive, partial, potential (based on variants of unknown significance), or unknown. The examined cohort was predominantly pediatric, the most common clinical indications were neurological, dysmorphic, and metabolic disorders. Definitive molecular diagnoses were established in 24.8 % of patients. Inclusion of potential diagnoses increased the total yield to 48.6 %. Diagnostic efficacy varied significantly among disease categories ranging from 58.3 % for renal disorders to 0 % for neurodevelopmental disorders. A total of 420 unique variants were analyzed, and missense changes were the most frequent among clinically significant findings. The most commonly implicated genes were ATP7B, GJB2, ABCA4, and GALT. The study results indicate that WES is an effective first-tier molecular tool for a wide range of suspected hereditary diseases in the Yugra population, with a diagnostic yield comparable to similar studies abroad. The findings support the utility of WES in diverse populations and highlight the potential for increasing yield through trio-WES and periodic data reanalysis.

## Abstract (ru)

Полноэкзомное секвенирование произвело революцию в диагностике наследственных заболеваний, однако его эффективность варьирует в разных популяциях. Данные о генетической архитектуре редких наследственных заболеваний во многих регионах России, включая этнически разнообразный Ханты-Мансийский автономный округ – Югру, ограниченны. Настоящее исследование направлено на оценку диагностической ценности полноэкзомного секвенирования для выявления генетических вариантов, ассоциированных с наследственными заболеваниями в этой этнически неоднородной популяции. В исследование включили 286 пробандов с предполагаемыми наследственными заболеваниями, наблюдаемых генетиками региона в период с 2021 по 2024 г. Полноэкзомное секвенирование было выполнено на платформе DNBSEQ-G50 (MGI, Китай). Биоинформатический анализ включал выявление и аннотацию вариантов с использованием популяционных баз данных и инструментов прогнозирования патогенности. Найденные варианты были распределены по категориям в соответствии с рекомендациями ACMG/Российского общества медицинских генетиков и коррелировали с клиническими фенотипами. Молекулярно-генетические диагнозы были классифицированы как полные, частичные, потенциальные (на основании вариантов неизвестной значимости) или отсутствующие. Обследованная когорта преимущественно состояла из детей, наиболее частыми клиническими показаниями были неврологические, дисморфические и метаболические нарушения. Окончательный молекулярно-генетический диагноз был установлен у 24.8 % пациентов. С учетом потенциальных заключений общая значимость исследования увеличилась до 48.6 %. Диагностическая эффективность значительно варьировала в зависимости от категории заболеваний: от 58.3 % для заболеваний почек до 0 % для нарушений нейрогенеза. Проанализировано в общей сложности 420 уникальных вариантов генов, при этом миссенс-варианты были самыми встречающимися среди клинически значимых результатов. Наиболее часто выявлялись гены ATP7B, GJB2, ABCA4, GALT. Результаты исследования показали, что полноэкзомное секвенирование – эффективный молекулярный тест первой линии для широкого спектра предполагаемых наследственных заболеваний у жителей Югры с диагностической ценностью, сопоставимой с аналогичными исследованиями за рубежом. Полученные данные подтверждают результативность полноэкзомного секвенирования в гетерогенной популяции и подчеркивают потенциал повышения диагностической ценности за счет формата «трио» и периодического повторного анализа данных.

## Introduction

Due to the development of molecular genetic diagnostic methods, especially high-throughput sequencing, significant progress has been made in determining the molecular nature of hereditary diseases over the past 15 years. In particular, the introduction of whole-genome (WGS) and whole-exome (WES) sequencing has enabled both large-scale population projects to describe the frequencies of genetic variants and the analysis of complex clinical cases with unclear disease etiology.

Although technological advances allow for more precise results and cheaper research, the clinical interpretation of genomic data necessary for a specific patient has become a new challenge (Petersen et al., 2017). Given the enormous amount of data obtained from WGS, the main obstacles to implementing this method are the difficulty of interpretation in the context of a specific disease, the high cost, and the burden on laboratory infrastructure with the general diagnostic efficiency of about 40 % (Stranneheim et al., 2021).

In terms of diagnostic efficiency, WES allows focusing on the analysis of protein-coding regions of genes. It is hardly inferior to WGS, and its advantage is less laborious interpretation. However, its key disadvantages are the loss of information outside of exons, the uneven coverage of gene sequences by probes, and limitations in analyzing copy number variations (CNVs) (Wang et al., 2017; Ross et al., 2020).

Since the diagnostic efficiency of WES for hereditary diseases significantly exceeds that of targeted panels, its main niche includes cases with suspected rare genetic diseases, diseases with recently identified or extended genes, a suspected heterogeneous disease in a young child, and negative results from other diagnostic methods (Okuneva et al., 2020).

According to OMIM Morbid Map Scorecard (www.omim. org/statistics/geneMap, accessed September 25, 2025), there are currently 6,619 phenotypes with 4,661 involved genes corresponding to single gene disorders and traits with known molecular basis. Most common forms of monogenic diseases vary significantly not only among different countries but also within distinct regions of a country. Therefore, it is of utmost importance to study the spectrum of genetic variants and monogenic diseases in all regions of Russia (Zinchenko et al., 2019), taking into account the vast diversity of subpopulations that have been practically unexplored by large-scale studies (Barbitoff et al., 2024).

The aim of this study was to identify the genetic causes of the most common hereditary diseases in the West Siberian Russian Khanty-Mansi Autonomous Okrug (Yugrа) and to assess the diagnostic efficiency of WES in this population.

## Materials and methods

Patient enrollment. Patient selection was carried out from 2021 to 2024 inclusive, as they were admitted for consultation at the regional medical genetic service located in Surgut (Yugra). For the study, 286 probands of various ages and ethnicities were selected according to the following inclusion criteria: the suspected monogenic nature of the disease (early age of manifestation, indications in the family history, inherited nature of the disorder, rare and specific symptoms of multiple organ damage), as well as already diagnosed hereditary diseases with an unspecified molecular cause based on clinical picture and common biochemical tests. For molecular genetic testing, 5 mL of peripheral blood (with EDTA) were taken from patients and transferred for processing to the Yugra biobank laboratory, established at the Surgut State University. All participants (or their official representatives) provided informed consent for participation in the study and personal data processing. The study was conducted in accordance with the Helsinki Declaration

DNA extraction, library preparation, and sequencing. Genomic DNA was extracted from peripheral blood using a MagPure Blood DNA Kit (Magen, China). Whole-exome libraries were prepared using a KAPA HyperPlus Kit and KAPA HyperExome probes (Roche, United States). The libraries were converted with a MGIEasy Universal Library Conversion Kit (MGI, China) and sequenced on a DNBSEQ-G50 system (MGI) in the paired-end mode with the read length of 150 bp, following the manufacturer’s recommendations.

Bioinformatics analysis. Samples with an average 70× coverage of target regions and at least 10× coverage width of 98 % were included in further analysis. Samples that did not pass quality control were sent for repeated library preparation and sequencing.

Mapping of obtained reads to the human reference genome (hg19 in 2021–2022 and hg38 in 2023–2024) was performed using BWA (0.7.16) (Li, 2011). Post-processing steps of alignments, variant calling, and filtering were carried out using the Genome Analysis Toolkit (Van der Auwera, O’Connor, 2020).

Variant annotation for all known transcripts of each gene from the RefSeq database was performed using snpEff (v.5.1) (Cingolani et al., 2012), with population frequencies of identified variants from The 1000 Genomes Project and gnomAD samples added to the annotation. Pathogenicity prediction of sequence variants was performed using DANN (Quang et al., 2015), GERP (Davydov et al., 2010), REVEL (Ioannidis et al., 2016), SIFT (Kumar et al., 2009), PolyPhen2 (Adzhubei et al., 2010), PrimateAI (Sundaram et al., 2018). Algorithms AdaBoost (Pashaei et al., 2016) and SpliceAI (Strauch et al., 2022) were also used for assessing the impact of variants on the splice site function.

Clinical data interpretation. To assess the clinical relevance of identified sequence variants the following resources were used: OMIM database, disease-specific databases (if available), and scientific literature data. Reports included only variants that had a possible relationship to the patient’s clinical manifestations or met other criteria specified in the physician’s referral. Polymorphisms classified as benign or likely benign were excluded from the report.

Identified variants were categorized according to criteria from ACMG guidelines (Richards et al., 2015) and the Russian Society of Medical Genetics (Ryzhkova et al., 2019) as pathogenic (P), likely pathogenic (LP), variants of unknown (clinical) significance (VUS), and asymptomatic carriers (AC). The last category included heterozygous pathogenic and likely pathogenic variants not causing the disease in the proband but strongly associated with other monogenic diseases.

Classification of molecular diagnoses. Based on the number of identified variants in each gene and inheritance type, molecular testing results were categorized as follows:

– complete molecular genetic diagnosis (MGD): at least one heterozygous or hemizygous P/LP variant with a dominant or X-linked type of inheritance, as well as one homozygous P/LP variant or two (potentially) compound heterozygous P/LP variants with a recessive type of inheritance; – partial MGD: one heterozygous P/LP variant in a gene associated with an autosomal recessive disease; – potential MGD: one VUS clearly associated with the phenotype with autosomal dominant or X-linked inheritance, or two VUSes clearly associated with the phenotype with autosomal recessive inheritance; – no MGD: absence of identified variants, one P/LP variant or VUS in a gene with autosomal recessive inheritance, incomplete relation of VUS associated with autosomal dominant, or X-linked inheritance to clinical presentation; – incidental findings: only AC variants. Statistical analysis and graph plotting were performed using the RStudio programming environment. Patient data and genetic variants were imported from a proprietary database (Glotov et al., 2025). They are available upon request.

## Results

**Characteristics of the examined cohort of patients**

From 2021 to 2024 inclusive, 286 probands with suspected genetic diseases were referred from the regional medical genetics counseling service for whole-exome sequencing. The gender, age, and ethnic compositions of the subjects are presented in Table 1. The majority of patients (86.7 %) belong to the child-adolescent age group, the mean age of the cohort being 10.9 years. The most represented ethnic groups are Russians (65.4 %) and Tatars (11.5 %).

**Table 1.**
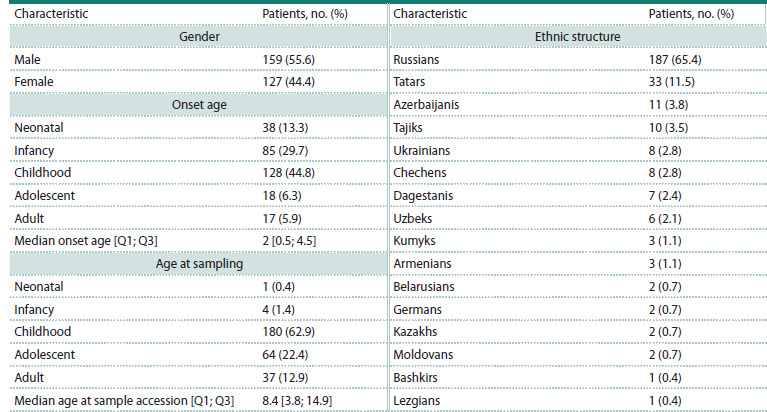
Demographic characteristics of the studied cohort (N = 286)

Regarding the structure of symptom categories (Table 2), neurological disorders occupy the leading position in Yugra (41.3 %). They are followed by dysmorphic syndromes (12.2 %) and metabolic disorders (11.2 %). It should be noted that psychiatric disorders are the only category not represented in the study group.

**Table 2.**
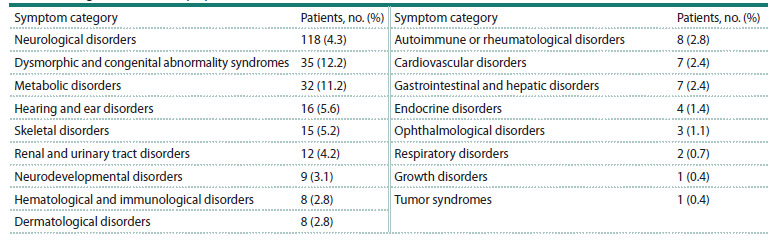
Сategories of clinical symptoms (N = 286)

**Clinical and molecular characteristics of identified genetic variants related to the phenotype**

**Fig. 1.**
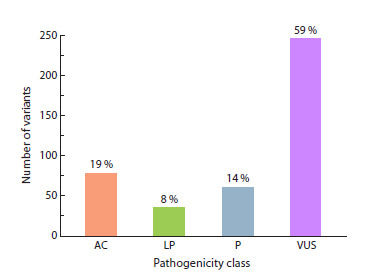
Pathogenicity classes of the identified variants. AC – asymptomatic carrier; LP – likely pathogenic; P – pathogenic; VUS – variant of unknown significance.

A total of 420 genetic variants (SNVs, InDels) were identified in patients. These variants were associated either with the observed phenotype or with the asymptomatic heterozygous carrier status (AC) of various monogenic diseases. Although, as shown in Figure 1, most of the identified variants are not of confirmed clinical significance, their potential contribution to disorder manifestation cannot be ignored.

**Fig. 2.**
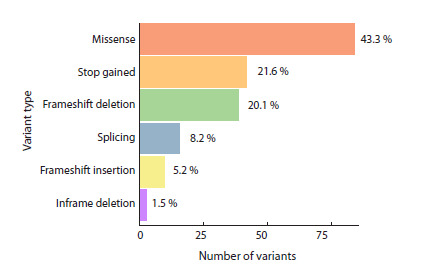
Molecular classes of clinically significant variants

The clinically significant variants detected (194 out of 420) (Fig. 2) belong to the following classes: missense, 43.3 %; stop-gained, 21.6 %; frameshift deletions, 20.1 %; splice donor/ acceptor variants, 8.2 %; frameshift insertions, 5.2 %; inframe deletions, 1.5 %. It should be noted that the class of repeat length variation is not represented in our study, as the bioinformatics algorithm used has not undergone strict validation for the analysis of this variation type.

**Fig. 3.**
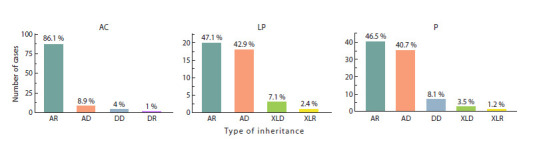
Distribution of pathogenicity classes by inheritance types. AR – autosomal recessive; AD – autosomal dominant; DD – digenic dominant; DR – digenic recessive; XLD – X-linked dominant; XLR – X-linked recessive.

The inheritance patterns of identified clinically significant variants are presented in Figure 3. Gene variants with the autosomal recessive inheritance pattern have a significant weight in the cohort studied (47.6 % for LP and 46.5 % for P). Autosomal dominant inheritance is observed for a substantial number of P/ LP variants (40.7 and 42.9 %, respectively). X-linked inheritance has a relatively low prevalence in the cohort, accounting for no more than 10 % of all likely pathogenic variants and no more than 5 % of all pathogenic variants

**Fig. 4.**
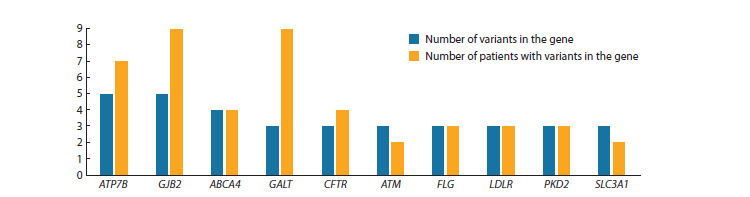
Human genes with the largest numbers of variants in the studied cohort.

Among the genes with the greatest number of detected variants, as shown in Figure 4, the most prominent are ATP7B, GJB2, ABCA4, and GALT, which are associated with Wilson’s disease, GJB2-related deafness, Stargardt disease, and galactosemia, respectively

The most frequent clinically significant variant, as presented in Table 3, is c.3207C>A in the ATP7B gene, associated with Wilson’s disease. However, the majority of the most frequent variants were detected in the AC (asymptomatic carrier) state. All most frequent variants detected are described in the literature as pathogenic, including the CFTR c.274G>A (p.Glu92Lys) variant (Chuvash mutation), which is absent from the gnomAD database.

**Table 3.**
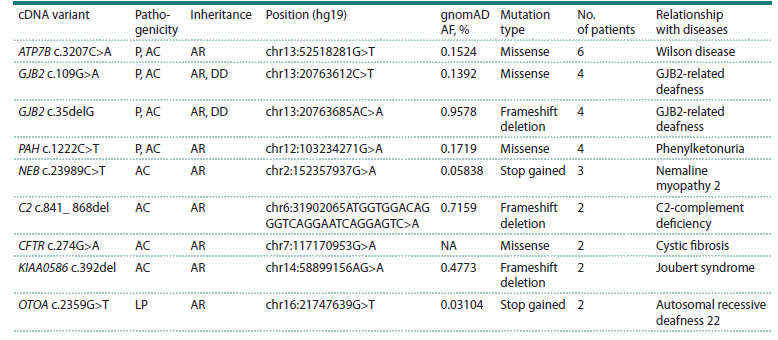
The most common clinically significant variants in the studied cohort

**Clinical effectiveness of WES in the examined cohort**

**Fig. 5.**
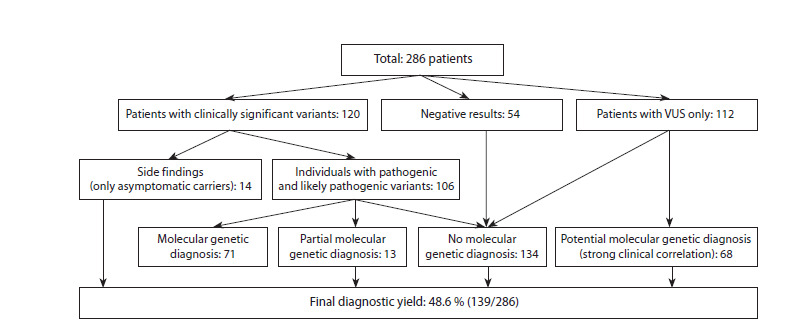
WES testing summary.

As shown in Figure 5, WES results produced no findings in 54 out of 286 examined patients. Variants of P/LP and AC classes were found in 120 patients, with 14 of them having only AC as a secondary finding. Among 106 patients with phenotype-relevant variants, 71 received a molecular genetic diagnosis, and 13 had only a partial diagnosis. Among the 112 studied samples with only VUS, 68 patients had variants that allowed for a potential molecular genetic diagnosis. Thus, the diagnostic effectiveness of WES in the examined cohort was 48.6 % (139/286).

**Fig. 6.**
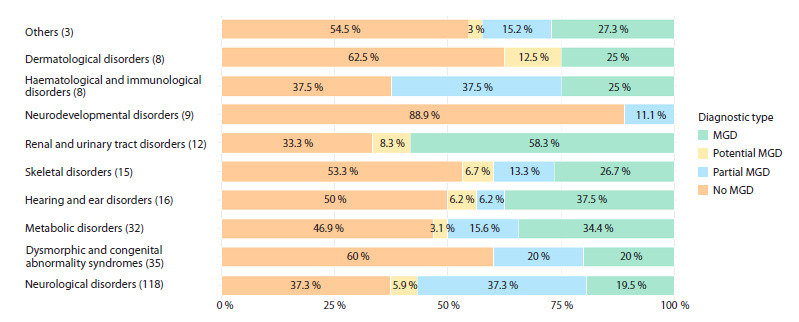
Types of molecular genetic diagnosis (MGD) in the most common disorder groups.

The distribution of molecular genetic diagnosis types varies significantly across different symptom categories (Fig. 6). The highest percentages of patients with confirmed molecular genetic diagnosis belong to the following categories: Renal and urinary tract disorders, 58.3 % (7/12 patients); Hearing and ear disorders, 37.5 % (6/16 patients), and Metabolic disorders, 34.4 % (11/32 patients). The lowest diagnostic effectiveness among the most common categories is observed in the Neurodevelopmental disorders group, where 88.9 % of patients (8/9) received no molecular genetic diagnosis.

The most ambiguous molecular diagnoses are observed in Haematological and immunological disorders – 37.5 % with potential MD (3/8 patients) and Neurological disorders – 37.3 % with potential MD (44/118 patients). The presence of a large number of variants of uncertain clinical significance (VUS) explains the ambiguity in these groups.

## Discussion

We performed whole-exome sequencing for 286 probands with suspected hereditary diseases to clarify the molecular genetic diagnosis. The majority of patients (86.7 %) belonged to the child-adolescent age group, which is associated with the early manifestation of hereditary diseases. Male subjects were more numerous in the group studied (55.6 %), owing to the presence of X-linked recessive disorders.

The diagnostic effectiveness of the method was 24.8 % for cases with complete molecular genetic diagnosis and increased up to 48.6 % when taking into account variants of uncertain significance that have a close relationship with the observed phenotype. These variants may be reclassified using updated information in the foreseeable future, as with similar findings from studies in other countries (Han et al., 2025).

As long as WES testing was conducted only for probands, the experience of other researchers brought us to the assumption that diagnostic effectiveness could be increased significantly through the implementation of duo- and trio-WES (Lai et al., 2024), mainly due to more reliable identification of compound heterozygous variants and de novo variants (Tan et al., 2019). This is particularly relevant for the studied population, where the proportion of autosomal recessive pathogenic or likely pathogenic variants is close to 50 %

Additionally, diagnostic effectiveness could be improved through re-analysis of exome data due to the constant emergence of new data about the relationships between variants and phenotypes (Arteche-López et al., 2022). Finally, since approximately 13 % of genetic variability is associated with copy number variations (CNVs) (Stankiewicz, Lupski, 2010), we conceive that it would be desirable for patients with negative WES results to undergo whole-genome sequencing (WGS) or low-coverage genome sequencing in combination with re-analysis of WES (Moey et al., 2025).

We found that the most common symptom categories of hereditary diseases in Yugra were Neurological disorders (118 out of 286 cases), Dysmorphic and congenital abnormality syndromes (35/286); and Metabolic disorders (32/286).

Our study shows significant differences in WES diagnostic effectiveness among different symptom categories: from zero yield for percentage of confirmed molecular genetic diagnosis cases in the Neurodevelopmental disorders group to 58.3 % in the Renal and urinary tract disorders group. Thus, the high occurrence of neurological disorders in our cohort presumes that just nervous system disorders pose the greatest challenge to productive WES analysis. Such differences may be related to yet uncharacterized molecular causes of these diseases (Adams, Eng, 2018), and generally they are consistent with results obtained from the analysis of 3,040 WES samples, where a wide range from 4 to 55 % of positive reports (Retterer et al., 2016) was also observed in different disease groups.

It is also important to consider the relevance of applying special molecular genetic methods to specific inherited diseases, such as search for duplications on chromosome 17 in cases of hereditary motor-sensory neuropathies (Shchagina et al., 2020), which was not conducted within the scope of our study. Other studies note the effectiveness of duo- and trio- WES for patients with intellectual disability, developmental delays, and epilepsy due to the high number (up to 80 %) of de novo variants in these cases (Lai et al., 2024).

It is particularly important to emphasize the significance of providing sufficient data on clinical picture by referring physicians (mostly by geneticists), as incomplete understanding of the symptoms by the interpreter may lead to false elimination of many genes from the search criteria and produce false-negative results. Additionally, for multiethnic populations determining the patient’s ethnicity (at least based on a questionnaire) is an important aspect, which was considered in our study, involving 16 ethnic groups.

Furthermore, the importance of secondary findings classified in our study as the asymptomatic carrier state (about 20 % of all identified variants) should not be underestimated, as they provide insight into the genetic burden of the population and should be appropriately considered during genetic counseling of families, particularly for planning future pregnancies.

It can be noted that 8.9 % of asymptomatic carrier cases among the examined patients are referred to genes with an autosomal dominant inheritance pattern. However, for these cases no significant effect on the phenotype was observed, which may be due to the different inheritance patterns of the variant depending on its belonging to a particular protein domain, as in the case of GJB2 (Xiang et al., 2023). Another consideration is the risk of mutation manifestation at an older age, as in the case of mutations in the BRCA1 and BRCA2 genes (Azzollini et al., 2016).

## Conclusion

In our study of 286 residents of the KHMAO-Yugra region, we accurately established molecular genetic diagnoses in 24.8 % of patients by using the whole-exome sequencing approach and identified the possible genetic nature of inherited diseases in 48.6 % of cases within the ethnically heterogeneous group. We characterized 420 gene variants, excluding benign and likely benign ones. Thus, whole exome sequencing should be considered a sufficiently effective first-tier diagnostic method for unveiling the genetic causes of a broad range of presumably hereditary diseases. However, some specific cases may demand duo- or trio-WES, whole-genome sequencing, or additional specific methods well proven in revealing the molecular background of certain classes of molecular disorders

## Conflict of interest

The authors declare no conflict of interest.
